# R1507, an Anti-Insulin-Like Growth Factor-1 Receptor (IGF-1R) Antibody, and EWS/FLI-1 siRNA in Ewing's Sarcoma: Convergence at the IGF/IGFR/Akt Axis

**DOI:** 10.1371/journal.pone.0026060

**Published:** 2011-10-11

**Authors:** Helen J. Huang, Laura S. Angelo, Jordi Rodon, Michael Sun, Klaus-Peter Kuenkele, Henrique A. Parsons, Jonathan C. Trent, Razelle Kurzrock

**Affiliations:** 1 Phase I Program, Department of Investigational Cancer Therapeutics, The University of Texas M.D. Anderson Cancer Center, Houston, Texas, United States of America; 2 Servei d'Oncologia Medica, Vall d'Hebron Institute of Oncology, Hospital Universitari Vall d'Hebron, Barcelona, Spain; 3 Roche Diagnostics GmbH, Penzberg, Germany; 4 Division of Cancer Medicine, Department of Sarcoma Medical Oncology, The University of Texas M.D. Anderson Cancer Center, Houston, Texas, United States of America; University of Pittsburgh, United States of America

## Abstract

A subset of patients with Ewing's sarcoma responds to anti-insulin-like growth factor-1 receptor (IGF-1R) antibodies. Mechanisms of sensitivity and resistance are unknown. We investigated whether an anti-IGF-1R antibody acts via a pathway that could also be suppressed by small interfering (si) RNA against the EWS/FLI-1 fusion protein, the hallmark of Ewing's sarcoma. The growth of two Ewing's sarcoma cell lines (TC-32 and TC-71) was inhibited by the fully human anti-IGF-1R antibody, R1507 (clonogenic and MTT assays). TC-32 and TC-71 cells express high levels of IGF-2, while RD-ES and A4573 Ewing's cell lines, which were less responsive to R1507 in our assays, express low or undetectable IGF-2, respectively. TC-71 cells also expressed high levels of IGF-1R, and R1507 decreased steady-state levels of this receptor by internalization/degradation, an effect which was associated with a decrease in p-IGF-1R, p-IRS-1, and p-Akt. EWS/FLI-1 siRNA also decreased p-Akt, due to its ability to increase IGF-BP3 levels and subsequently decrease IGF-1 and IGF-2 levels, thus inhibiting signaling through p-IGF-1R. This inhibition correlated with growth suppression and apoptosis. The attenuation of Akt activation was confirmed in TC-71 and HEK-293 (human embryonic kidney) cells by transfecting them with IGF-1R siRNA. We conclude that antibodies and siRNA to IGF-1R, as well as siRNA to EWS/FLI-1, act via intersecting IGF/IGF-1R signals that suppress a common point in this pathway, namely the phosphorylation of Akt.

## Introduction

Ewing's sarcoma is an aggressive small round blue-cell tumor that arises in the bone and soft tissue of young patients [1], [2]. Eighty-five percent of Ewing's sarcomas contain the t(11;22)(q24;q12) reciprocal chromosomal translocation, which results in the generation of a novel fusion protein combining the N-terminal transactivation domain of the Ewing's sarcoma breakpoint region 1 gene (EWSR1) and the C-terminal DNA binding domain of an ETS (E26 transformation-specific) family member gene [3], most commonly the FLI-1 (Friend leukemia integration 1) transcription factor gene. EWS/FLI-1 is a potent transcription factor and has been shown to both repress and activate specific target genes and function as an oncoprotein [3].

Therapy of Ewing's sarcoma includes surgery, radiation, and systemic chemotherapy in various combinations [1]. A subset of patients with Ewing's sarcoma have remarkable responses to insulin-like growth factor-1 receptor (IGF-1R) inhibitors [4]–[9], including the fully human anti-IGF-1R antibody, R1507 [4]. Why some patients respond, and what factors underlie resistance remains unclear. Preclinical studies suggest that levels of the IGF/IGFR machinery might be a factor in response and resistance [10]. We therefore characterized the status of various molecules important in the IGF signaling pathway in four Ewing's sarcoma cell lines, investigated their response to the anti-IGF-1R antibody R1507, and explored the relationship between the EWS/FLI-1 fusion protein and the IGF machinery, including signaling molecules downstream of the IGF-1R in Ewing's sarcoma. We demonstrate that the anti-IGF-1R antibody (R1507) and small interfering RNA (siRNA) against the Ewing's fusion protein (EWS/FLI-1) suppress a common pathway involving the IGF/IGFR/Akt axis through inhibition of different targets in the IGF/IGF-1R cascade.

## Materials and Methods

### Cell lines

RD-ES is a Ewing's sarcoma cell line obtained from American Type Culture Collection (ATCC). A4573, TC-32, and TC-71 were a generous gift of Dr. Jeff Toretsky [11]. HEI-193 (human schwannoma), SK-N-AS (human neuroblastoma from ATCC), HEK 293 (human embryonic kidney from ATCC), and MCF7 (human breast cancer from ATCC) cell lines were used as non-Ewing's sarcoma controls. The HEI-193 cell line was generously provided by the House Ear Institute [12]. All cell lines were maintained in RPMI-1640 (GIBCO/BRL) or DMEM plus 10% fetal calf serum (FCS) and maintained in a 37°C incubator with 5% CO_2_.

### Reverse Transcription-Polymerase Chain Reaction (RT-PCR)

RT-PCR was performed to confirm the type of EWS/FLI-1 fusion protein in the Ewing's sarcoma cell lines. Briefly, RNA was extracted from cell lines using the RNeasy™ mini kit (Qiagen) and used for reverse transcription followed by PCR using primers (Sigma-Genosys) as described [13]–[15]. PCR products were run on agarose gels, stained with ethidium bromide, and photographed (Alpha Innotech).

### Sequencing of IGF-2R ligand binding domain

To analyze IGF-2R polymorphisms, genomic DNA was extracted from Ewing's sarcoma cell lines using the QIAmp mini-prep kit (Qiagen). PCR primers were designed for exons 27–40 (IGF-2 ligand binding domain) of IGF-2R (SeqWright, Inc., Table S1). PCR reactions were conducted with HotStar HiFidelity PCR Kit (Qiagen) according to the manufacturer's protocol. PCR reactants were purified with QIAquick spin columns (Qiagen) and sent to SeqWright, Inc. for sequencing. Other regions of the IGF-2R gene were also studied [16]. Polymorphisms were detected by comparing generated DNA sequences to reference sequences (NM_000876.2, NT_007422.13, and NW_001838991.2) using the web-based software blast.ncbi.nlm.nih.gov/Blast.cgi.

### Antibodies and Reagents

R1507, a fully human IgG_1_ monoclonal antibody to IGF-1R, (Roche Diagnostics, Penzberg, Germany), was diluted in medium immediately before use. Recombinant (r) IGF-BP3 and rIGF-1 were purchased from Sigma. Primary antibodies included rabbit polyclonal antibody against ERK1/2, p-ERK1/2, IGF-1Rβ, phosphorylated (p)-IGF-1R (Tyr 1135/1136), Akt, and p-Akt, (ser 473) (Cell Signaling), anti -IRS-1 and -p-IRS-1 (Tyr 612) (Upstate Cell Signaling Solutions), anti-EWS, -IGF-2, -IGF-2R, and -GAPDH (Santa Cruz Biotechnology). Secondary antibodies for Western blot included anti-rabbit, anti-mouse, (BioRad), and goat anti-human conjugated to horse radish peroxidise (HRP) (Millipore-Chemicon).

### Western blot analysis

Cells were seeded in six well plates and treated as described in the figure legends. For cytoplasmic proteins, cells were washed with PBS and lysed in 1× RIPA buffer (Millipore) plus protease inhibitors. For EWS/FLI-1, nuclear extracts were obtained by incubating for 15 minutes in lysis buffer (0.3% NP-40) followed by 30 minutes in ice-cold extraction buffer plus protease inhibitors. A modified Lowry protein assay was performed (Pierce Biotechnology), and equal amounts (20–50 µg) of protein were loaded onto SDS-PAGE gels. Proteins were transferred to nitrocellulose membranes by electro-blotting, incubated with primary antibody at room temperature for two hours, or at 4°C overnight, washed, and then incubated with secondary antibody conjugated to HRP and detected by enhanced chemiluminescence (Amersham Pharmacia Biotech). Blots were exposed to film and densitometry was performed using a Hewlett Packard scanner and the NIH Image software (Scion Corporation).

### ELISA

Enzyme-linked immunosorbent assays (ELISAs) included free IGF-1 and IGF-BP3 (R&D Systems), and IGF-2 (Diagnostic Systems Laboratories). Cells were seeded in six well plates for 24 or 48 hours, supernatants were collected, and ELISA was performed. The mean lower limits of sensitivity were 0.026 ng/ml and 2.2 ng/ml for the IGF-1 and IGF-2 ELISAs, respectively. Because of the low concentration of IGF-BP3 in the Ewing's cell lines, supernatants were concentrated 40× using Amicon centrifugal filter units (Millipore) prior to ELISA. The mean lower limit of sensitivity for this assay was 0.05 ng/ml. Absorbance was read using a plate reader (Molecular Devices) at 570 nm.

### Clonogenic assay

Clonogenic assays were performed to determine the effect of R1507 antibody and EWS/FLI-1 siRNA on Ewing's sarcoma cell growth. 1×10^3^ cells per well of a 6 well plate coated with 1% gelatin were seeded in media containing 10% FCS, and either treated with a single dose of 1–50 µg/ml R1507 or transfected with EWS/FLI-1 siRNA and allowed to grow for 1 week. Colonies were counted and compared to untreated control dishes, which were assigned a value of 100% growth.

### MTT assay

Survival of the cells was assessed by MTT (3-[4,5-dimethylthiazol-2-yl]2,5-diphenyltetrazolium bromide) assay, according to the manufacturer's instructions (Sigma-Aldrich). After 48 hours of incubation, conversion of MTT was measured at 570 nm and is expressed as the change in cell growth as compared to untreated (control) cultures, which were assigned 100% growth.

### Annexin V apoptosis assay

Apoptosis of Ewing's sarcoma cells was measured by staining cell-surface phosphatidyl serine with FITC-conjugated Annexin V. 5×10^5^ (R1507 treated) and 2.5×10^5^ cells (siRNA transfected) were seeded per well of a 96 well plate and treated for 48, 72, or 96 hours, harvested, and stained with FITC-Annexin V and propidium iodide according to the manufacturer's instructions (BD Pharmingen). Cells were analyzed by fluorescence activated cell sorter (FACS) analysis (Becton Dickinson, Bedford, MA).

### siRNA transfection

2.5×10^5^ TC-71 or HEK 293 cells were seeded per well of a 6 well plate and transfected with DharmaFECT 1 siRNA lipid transfection reagent alone (Thermo Scientific Dharmacon), non-targeted siRNA (siGENOME non-targeting siRNA #1), human EWS/FLI-1 (EWSR1) siRNA, IGF-1R siRNA, or GAPDH siRNA (control) for 48 hours. Following transfection, cell lysates and culture supernatants were harvested for Western blot analysis and ELISA, respectively.

### Statistical analyses

Descriptive statistics were used to summarize the data. Statistical significance of the differences between continuous variables was calculated using Student's t tests or Mann-Whitney tests, depending on the normality of the data. Comparisons between three or more groups were analyzed for statistical significance using an analysis of variance test (one-way ANOVA) with contrast between pairs of groups when appropriate. P values lower than 0.05 were considered statistically significant. Analyses were made using SPSS version 16 (SPSS Inc., Chicago, IL).

## Results

### Confirmation of EWS/FLI-1 fusion protein type

**Figure 1:**
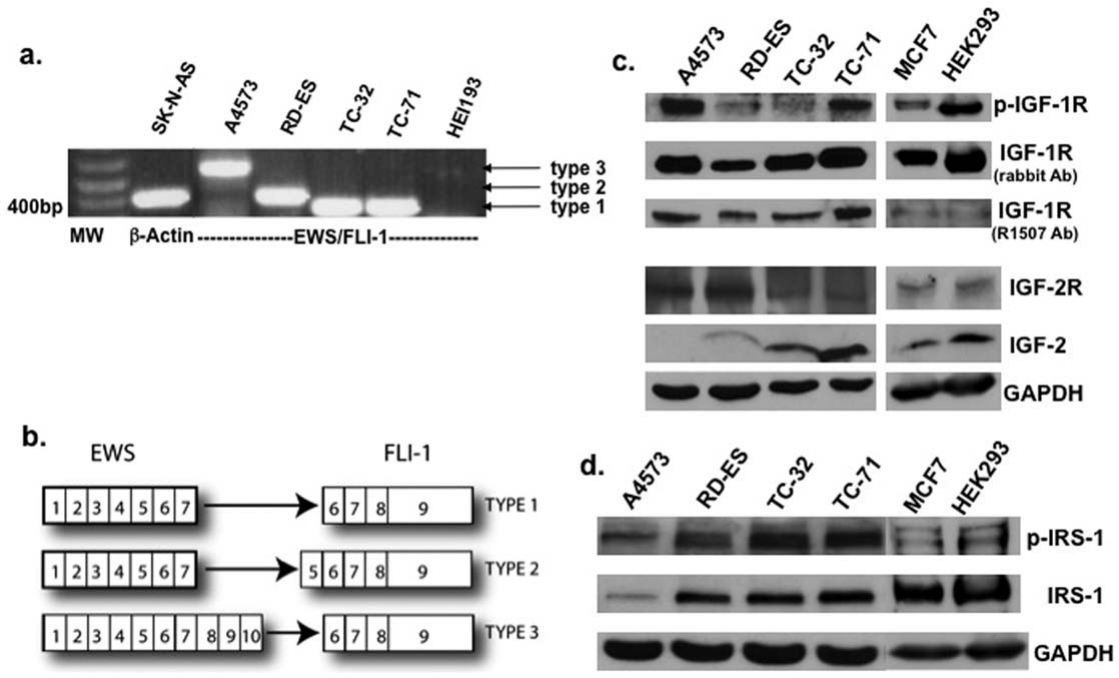
EWS/FLI-1 fusion protein type and baseline expression levels of IGF-1 signaling molecules in Ewing's sarcoma and control cell lines. (a) RT-PCR of the EWS/FLI-1 fusion protein transcripts was performed using mRNA from four different Ewing's sarcoma cell lines (A4573, RD-ES, TC-32, and TC-71) and two control cell lines. Lane 1: DNA molecular weight marker (MW). Lane 2: amplification of β-actin using SK-N-AS mRNA. Lanes 3–6: RT-PCR with specific primers for the EWS/FLI-1 translocation t(11;22)(q24;q12). Lane 7: HEI-193 is negative for the fusion protein. Sizes for EWS/FLI-1 transcripts were: 327 base pairs (bps) for type 1; 422 bps for type 2; and 540 bps for type 3. (b) Schematic showing exon joining of the EWS gene with the FLI-1 gene to generate three different EWS/FLI-1 fusion proteins. (c) Baseline expression of IGF signaling molecules in Ewing's sarcoma and control cell lines by Western blot analysis. Baseline IGF-1R levels were analyzed using either a rabbit polyclonal antibody or the human R1507 anti-IGF-1R antibody. P-IGF-1R and IGF-2R were analyzed using appropriate antibodies. IGF- 2 levels in the culture supernatant were also analyzed by Western blot. Anti-GAPDH was used as a loading control. MCF7 and HEK293 cells were used as positive controls for IGF family member expression. (d) Steady state levels of p-IRS-1 and total IRS-1 in Ewing's sarcoma cell lines. Membranes were probed using anti-p-IRS-1, anti-IRS-1, or anti-GAPDH as a control.

A4573, RD-ES, TC-32, and TC-71 Ewing's sarcoma cell lines all contain the EWS/FLI-1 translocation t(11;22)(q24;q12). RT-PCR confirmed that A4573 expresses the type 3 fusion protein, RD-ES expresses type 2, and TC-32 and TC-71 express type 1 (Figure 1a) [17]–[19]. Amplification of messenger RNA from the SK-N-AS (neuroblastoma) cell line using β-actin primers was used as a positive control for the RT-PCR reaction (Figure 1a, lane 2). HEI-193 (human schwannoma) was used as a non-Ewing's sarcoma control and contains no EWS/FLI-1 fusion protein (Figure 1a lane 7). Figure 1b shows the different types of fusion proteins predicted from variable exon joining of the EWS and FLI-1 genes. All four cell lines expressed the EWS/FLI-1 fusion protein at similar levels by Western blot (data not shown).

### Baseline IGF-1R, p-IGF-1R, IRS-1, and p-IRS-1 expression levels

In order to determine if there was a correlation between IGF family member expression with response to anti-IGF-1R antibody treatment, Western blot and ELISA was performed to quantify the relative expression of these molecules at baseline. IGF-1R is a tyrosine kinase receptor that binds IGF-1 and -2 with high affinity. Baseline expression levels of IGF-1R and p-IGF-1R were examined by Western blot (Figure 1c). Expression of total IGF-1R was highest in TC-71 and A4573, followed by TC-32 and RD-ES cells, as determined by densitometry. A4573 and TC-71 cells expressed the highest levels of p-IGF-1R (Figure 1c). Insulin receptor substrate protein-1 (IRS-1) is an important signaling molecule downstream of IGF-1R, and its phosphorylation has been implicated in increased cell proliferation and survival via the phosphoinositide-3 kinase (PI3K)/Akt pathway [20], [21]. RD-ES, TC-32, and TC-71 expressed similar baseline levels of total IRS-1, while A4573 contained the least (Figure 1d). TC-32 and TC-71 expressed the most p-IRS-1 followed by RD-ES, then A4573. Note that the level of p-IGF-1R and p-IRS-1 does not always correlate within individual cell lines (Figure 1c and d). MCF7 and HEK293 cells were used as positive control cell lines for expression of IGF family members.

### IGF-2 levels, IGF-2R expression, and IGF-2R gene polymorphisms in the IGF-2 binding domain

The IGF-2/mannose-6-phosphate receptor (IGF-2R/M6PR) binds and internalizes IGF-2, thereby attenuating IGF-2's ability to signal through IGF-1R, the primary signaling receptor. Mutations in IGF-2R have been found in many types of cancer (see Discussion), and may predict response to IGF-1R inhibitors. Certain mutations reduce the amount of IGF-2R expressed on the cell surface, and increase the amount of free IGF-2, thereby allowing enhanced signaling through IGF-1R. IGF-2R expression was lower in TC-32 and TC-71 cells compared with A4573 and RD-ES cells. This may partially account for the higher levels of IGF-2 seen in the culture supernatant of these cells (Figure 1c).

A4573 cells contain the G>A single nucleotide polymorphism (SNP) (G**A**) at nucleotide position 5004 in the IGF-2R cDNA, which results in an arginine at amino acid position 1619 of the IGF-2 binding domain. These cells also expressed the lowest level of IGF-2 (Figure 1c). TC-32, TC-71, and RD-ES, which all express IGF-2, contain the A>G SNP (G**G**) at nucleotide position 5004 of IGF-2R, which corresponds to a glycine at amino acid position 1619 (Table 1). This SNP was previously studied in osteosarcoma [22], but had not been examined in Ewing's sarcoma. We did not detect any of the mutations previously associated with increased IGF-2 expression and response to IGF-1R inhibitors in the cell lines examined.

**Table 1:** Characteristics of Ewing's sarcoma cell lines.

	A4573	RD-ES	TC-32	TC-71
p-IGF-1R level*	++++	++	++	++++
IGF-1R level*	+++	++	++	+++
IGF-2R level*	++++	++++	++	+++
IGF-2R polymorphism	Exon 34	Exon 34	Exon 34	Exon 34
	nucleotide 5004	nucleotide 5004	nucleotide 5004	nucleotide 5004
	GA, Arg	GG, Gly	GG, Gly	GG, Gly
IGF-1 (ng/ml)(supernatant)	0.35	0.609	0.539	1.603
p-IRS-1 protein level*	++	+++	+++++	++++
IRS-1 protein level*	+	+++	++++	++++
IGF-BP3 (ng/ml)	0.81	0.72	1.0	0.15
IGF-2*	−	+	++	++++
EWS/FLI-1 fusion protein	Type 3	Type 2	Type 1	Type 1

*Based on densitometry of films, + = 5000 pixels.

### TC-71 cells express high levels of IGF-1 and low levels of IGF-BP3

**Figure 2:**
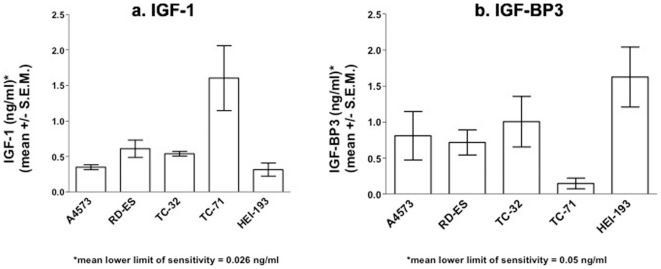
Baseline levels of IGF-1 and IGF-BP3 in the culture supernatants of four Ewing's sarcoma cell lines. (a) Baseline IGF-1 levels in the supernatant of Ewing's sarcoma and a control cell line. Cells were cultured at a density of 2–10×10^5^ per well of a six well plate. After 24 hours, supernatants were collected and IGF-1 levels were measured using an IGF-1 specific ELISA. Results show the mean and standard error of four experiments. (b) Baseline IGF-BP3 levels in the supernatant of Ewing's sarcoma and a control cell line. Cells were cultured as in (a), and supernatants were collected. Because of the low concentration of IGF-BP3 in the supernatant of Ewing's cell lines, supernatants were concentrated 40× prior to ELISA. Results show the mean and standard error of three experiments.

IGF-BP3 binds both IGF-1 and IGF-2. Most IGF in human serum is complexed to IGF-BP3 [20], [21]. IGF-BP3 inhibits the interaction of IGFs with IGF-1R. The amount of IGF-1 and IGF-BP3 secreted into the culture media by Ewing's sarcoma cell lines was measured by ELISA. TC-71 cells produced the most IGF-1 (1.603 ng/ml), followed by RD-ES, TC-32, and A4573 (Figure 2a and Table 1). [As noted above, TC-71 also had the highest level of IGF-2 (Figure 1c)]. HEI-193 schwannoma cells expressed IGF-1 levels comparable to A4573 cells. This result is not unexpected since many tumor cell lines produce IGF-1 *in vitro* and use IGF-1 or IGF-2 as autocrine growth factors [21]. Interestingly, TC-71 cells expressed the lowest amount of IGF-BP3 (0.15 ng/ml, Figure 2b), which may partially account for its elevated expression of IGF-1, IGF-2, and p-IGF-1R. The status of members of the IGF machinery in all four Ewing's cell lines are summarized in Table 1.

### The effect of R1507 anti-IGF-1R antibody on the proliferation of Ewing's sarcoma cell lines

**Figure 3:**
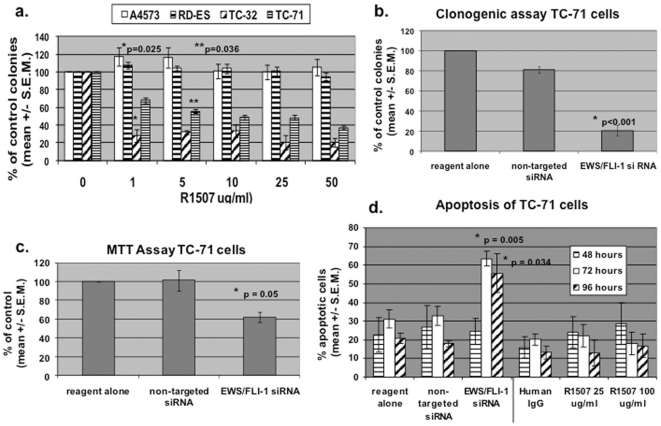
Inhibition of growth and induction of apoptosis in Ewing's sarcoma cell lines treated with R1507 or EWS/FLI-1 siRNA. (a) The effect of R1507 on the growth of four Ewing's sarcoma cell lines as assessed by clonogenic assay. Cells were treated with 1–50 µg/ml R1507 for one week. Colonies were counted and compared to control dishes (100% growth). Results show mean and standard error of 4 experiments. (b) TC-71 cell growth is inhibited by siRNA to the EWS/FLI-1 fusion protein. Cells were transfected with tranfection reagent alone, 50 nM non-targeted siRNA, or 50 nM EWS/FLI-1 siRNA once at the beginning of culture and allowed to grow for one week. Colonies were counted and compared to control dishes (non-targeted siRNA). Results show mean and standard error of three experiments. (c) The effect of EWS/FLI-1 siRNA on the growth of TC-71 cells as assessed by MTT assay. TC-71 cells were transfected with transfection reagent alone, 50 nM non-targeted siRNA, or 50 nM EWS/FLI-1 siRNA. After 48 hours, conversion of MTT was measured and is expressed as change in cell growth as compared to transfection reagent alone (100% growth). The mean and standard error of four experiments is shown. (d) Apoptosis of TC-71 cells following transfection with EWS/FLI-1 siRNA or treatment with R1507. TC-71 cells were either transfected with reagent alone, 50 nM non-targeted siRNA, or 50 nM EWS/FLI-1 siRNA. Alternatively, they were treated with human isotypic control IgG (100 µg/ml) or R1507 (25 or 100 µg/ml) for 48, 72, and 96 hours. Cells were stained with Annexin V-FITC and propidium iodide and analyzed by FACS analysis. The mean and standard error of three experiments is shown.

We examined the effect of R1507 on colony formation in Ewing's sarcoma cell lines by clonogenic assay (Figure 3a). No significant decrease in colony number was observed for A4573 and RD-ES Ewing's sarcoma cell lines, or for HEI-193 schwannoma cells (Figure 3a and data not shown). R1507 significantly decreased colony formation of TC-32 cells starting at 1 µg/ml (p = 0.025) and TC-71 starting at 5 µg/ml (p = 0.036) (Figure 3a). Inhibition of colony formation appears to be dose-dependent in TC-71 cells, whereas colony formation in TC-32 cells drops off rapidly at 1 µg/ml R1507. TC-71 cells were also treated with high dose (100 µg/ml) R1507 which resulted in a decrease in colony formation of 95% (data not shown).

### EWS/FLI-1 siRNA transfection decreases colony formation in TC-71 cells

Because TC-71 cells express the type 1 fusion protein, show a classic pattern of deregulation of molecules important in the IGF/IGFR axis (high IGF-1 and -2, low IGF-BP3, high IGF-1R and p-IGF-1R, high p-IRS-1, and low relative IGF-2R), and are sensitive to R1507 treatment in the clonogenic assay, they were chosen for further study. To evaluate the effect of blocking EWS/FLI-1 protein expression on cell growth, TC-71 cells were transfected with EWS/FLI-1 specific siRNA or non-targeted siRNA (Figure 3b). Down-regulation of EWS/FLI-1 fusion protein was confirmed by Western blot for each transfection. TC-71 colony formation significantly decreased following transfection with EWS/FLI-1 specific siRNA (p<0.001, Figure 3b). EWS/FLI-1 siRNA inhibited cell growth by 40% compared to cells transfected with non-targeted siRNA (MTT assay) (p = 0.05, Figure 3c). Two rounds of transfection with EWS/FLI-1 siRNA resulted in even greater inhibition of TC-71 cell growth by MTT assay (data not shown) [23].

### EWS/FLI-1 siRNA induces apoptosis of TC-71 cells, while R1507 does not

TC-71 cells were transfected with transfection reagent alone, non-targeted siRNA, or EWS/FLI-1 siRNA. Alternatively, cells were treated with human IgG, or two different concentrations of R1507. The Annexin V apoptosis assay was used to determine the percent of cells undergoing apoptosis. EWS/FLI-1 siRNA induced a significant increase in apoptosis, (27% and 38% at 72 (p = 0.005) and 96 hours (p = 0.034), respectively), compared to cells transfected with non-targeted siRNA (Figure 3d). In contrast, there was no significant increase in the percent of TC-71 cells undergoing apoptosis following treatment with either 25 or 100 µg/ml R1507 compared to a non-specific isotypic control (Figure 3d). Hence, EWS/FLI-1 siRNA induces apoptosis in TC-71 Ewing's sarcoma cells, while R1507 does not. This may be because EWS/FLI-1 siRNA has a wider range of effects than R1507, which inhibits cell growth mainly due to inhibition of proliferative pathways, rather than an increase in programmed cell death in this particular cell line.

### EWS/FLI-1 siRNA transfection increases IGF-BP3 and concomitantly decreases IGF-1 and IGF-2

**Figure 4:**
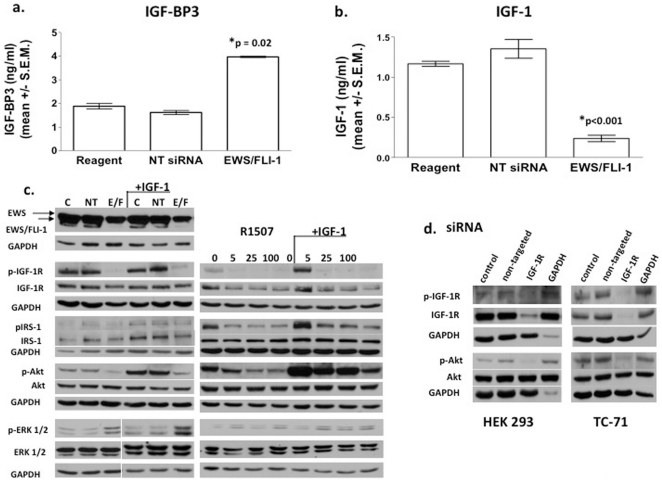
EWS/FLI-1 and R1507 influence the activation/expression of key molecules in the IGF-1 signaling pathway. (a) IGF-BP3 levels increase following transfection of TC-71 cells with EWS/FLI-1 siRNA. TC-71 cells were transfected with reagent alone, 50 nM non-targeted (NT) siRNA, or 50 nM EWS/FLI-1 siRNA for 48 hours. After three days total in culture, the supernatant was concentrated 20×. An ELISA for IGF-BP3 was performed. Results show the mean and standard error of four experiments. Starting amounts of IGF-BP3 are higher than those in Figure 2 because of the longer culture time required for transfection experiments. (b) IGF-1 levels decrease in EWS/FLI-1 siRNA-transfected TC-71 cell supernatant. TC-71 cells were transfected with 50 nM siRNA for 48 hours. Media was collected and concentrated 20×. IGF-1 ELISA was performed. Results show the mean and standard error of four experiments. (c) The effect of EWS/FLI-1 siRNA and R1507 on the expression of various molecules involved in IGF signaling pathways in TC-71 cells. Left-hand panel: TC-71 cells were transfected with reagent alone (C), 100 nM non-targeted siRNA (NT), or 100 nM EWS/FLI-1 siRNA (E/F) for 48 hours as described. Western blot analysis was performed using antibodies directed at molecules important in IGF signaling pathways. Anti-GAPDH was used as a loading control. Experiments were performed in the presence or absence of 50 µg/ml rIGF-1. Right-hand panel: TC-71 cells were treated with 0–100 µg/ml R1507 for 24 hours. Cells were harvested for Western blot and probed with relevant antibodies. Experiments were performed in the presence or absence of 50 µg/ml rIGF-1. (d) IGF-1R siRNA down-regulates total IGF-1R, p-IGF-1R, and p-Akt. HEK 293 and TC-71 cells were transfected with 100 or 50 nM siRNA against IGF-1R, respectively, for 48 hours. Cell lysates were harvested and Western blot was performed using antibodies to IGF-1R, p-IGF-1R, total Akt, and p-Akt (ser 473). SiRNA against GAPDH was used as a control.

IGF-BP3 increased 2-fold after EWS/FLI-1 siRNA transfection in TC-71 cells compared with non-targeted siRNA (p = 0.02, Figure 4a). This increase occurs presumably because EWS/FLI-1 represses transcription of IGF-BP3 at its promoter through direct DNA binding, and siRNA to EWS/FLI-1 blocks this repression [23]. A concomitant 5-fold decrease in the level of IGF-1 in the supernatant of TC-71 cells (p<0.001) occurs because of the increased level of IGF-BP3 available to bind IGF-1, and also because of decreased transactivation of the IGF-1 promoter by EWS/FLI-1 following transfection with EWS/FLI-1 siRNA (Figure 4a and b) [24]. IGF-2, which also binds IGF-BP3, decreases following transfection with EWS/FLI-1 siRNA as measured by ELISA (n = 12 repeats, p = 0.02, data not shown).

### EWS/FLI-1 siRNA transfection and R1507 down-regulate p-Akt in TC-71 cells

EWS/FLI-1 siRNA inhibited EWS/FLI-1 fusion protein expression by Western blot as expected (Figure 4c). Total IGF-1R levels remain unchanged, while p-IGF-1R decreased. P-IRS-1 decreases slightly after exposure to EWS/FLI-1 siRNA compared to non-targeted siRNA, while total IRS-1 levels remain unchanged. Akt (protein kinase B) is an important signaling molecule downstream of IGF-1R [20], [21]. Phosphorylation of Akt indicates the activation of growth stimulatory pathways by IGF. EWS/FLI-1 siRNA down-regulated p-Akt (ser 473), thus inhibiting IGF signaling (Figure 4c, left-hand panel).

R1507 down-regulated total IGF-1R levels because exposure to anti-IGF-1R antibody causes internalization and degradation of the receptor (Figure 4c, right-hand panel) [25], which results in decreased p-IGF-1R. Additionally, R1507 competes with IGF-1 and IGF-2 for binding to the IGF-1R, thus decreasing signaling (personal communication, K-P Kuenkele). R1507 also decreased p-IRS-1, and down-regulated Akt phosphorylation in a dose-dependent manner as a downstream effect resulting from the decrease in p-IGF-1R, while total IRS-1 and Akt levels remained unchanged. This inhibition appears to be competitive and concentration-dependent. Addition of 50 µg/ml rIGF-1 increased pretreated levels of p-IGF-1R, p-Akt, and p-IRS-1 (lanes 1&4, left panel, lanes 1&5, right panel, Figure 4c), indicating that this signal transduction pathway is active in TC-71 cells. rIGF-1 was unable to fully restore p-Akt or p-IGF-1R to pretreatment levels in the presence of EWS/FLI-1 siRNA or R1507 (lanes 1&6 left panel, lanes 1&8 right panel), but p-Akt and p-IGF-1R did increase slightly in the rIGF-1 plus EWS/FLI-1 siRNA or R1507 treated cells compared to cells treated with siRNA or R1507 alone (compare lanes 3&6 left panel and lanes 4&8, right panel, Figure 4c). The inability of R1507 or EWS/FLI-1 siRNA to completely abolish p-Akt, especially in the presence of rIGF-1, indicates that Akt is activated by other pathways in these cells, (e.g. epidermal growth factor receptor (EGFR) or insulin receptor (IR), and that a combination of agents, may further reduce activation of the Akt pathway [10], [26]–[28]. The MAP kinase pathway was not strongly activated in TC-71 cells, even in the presence of rIGF-1. Interestingly, R1507 treatment or EWS/FLI-1 siRNA transfection did not inhibit ERK 1/2 signaling, but rather resulted in increased ERK 1/2 phosphorylation (Figure 4c), perhaps because an alternative pathway to IGF was activated.

### IGF-1R siRNA down-regulates p-IGF-1R and p-Akt in TC-71 and HEK 293 (human embryonic kidney) cells

TC-71 and HEK 293 cells were transfected with IGF-1R siRNA to determine the effect of blocking IGF-1R expression on the phosphorylation of IGF-1R and Akt. Total IGF-1R levels were dramatically decreased, total Akt levels remained unchanged, while p-IGF-1R and p-Akt decreased in both cell lines (high IGF-1R expressers) (Figure 4d). Therefore, p-IGF-1R and p-Akt can be markedly down-regulated by decreasing cell-surface IGF-1R in TC-71 and HEK 293. Down-regulation of p-Akt by anti-IGF-1R antibodies occurs in cells of many different lineages, including small cell lung cancer (SCLC) [29], acute myeloid leukemia [30], and non-small cell lung cancer [31].

## Discussion

**Figure 5:**
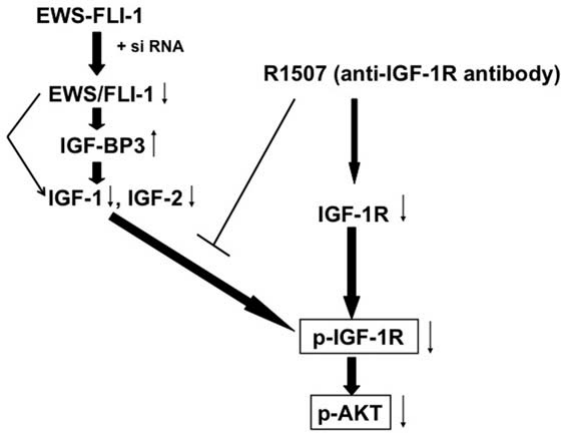
EWS/FLI-1 siRNA and R1507 block Ewing's sarcoma cell proliferation by inhibiting a shared pathway: phosphorylation of IGF-1R and Akt. EWS/FLI-1 siRNA increased IGF-BP3 while decreasing IGF-1. This results in decreased phosphorylation of IGF-1R and Akt. R1507 (anti-IGF-1R antibody) down-regulates cell-surface IGF-1R and competes with IGF-1 and -2 for binding to IGF-1R thereby inhibiting the phosphorylation of IGF-1R and Akt. Both EWS/FLI-1 siRNA and R1507 inhibit cellular proliferation by targeting a common pathway, namely the inhibition of p-IGF-1R and p-Akt (see text for details).

Following failure of front-line therapy, patients with metastatic Ewing's sarcoma have limited therapeutic options. Recent clinical trials have demonstrated that IGF-1R inhibitors can induce remarkable responses in some patients with advanced disease [4]–[9]. The molecular basis of these responses is unclear. The genetic hallmark of Ewing's sarcoma is the formation of the aberrant EWS/FLI-1 transcription factor. Our results suggest that EWS/FLI-1 siRNA and R1507, a fully human anti-IGF-1R antibody, inhibit a common IGF signaling pathway that converges on the suppression of p-Akt. SiRNA to EWS/FLI-1 accomplishes down-regulation of p-Akt by relieving transcriptional repression of IGF-BP3 and simultaneously inhibiting IGF-1 transcription [23], [24]. This results in a decrease in available IGF-1 (Figure 4b), which further decreases signaling through the IGF-1R (Figure 5). R1507 exerts its negative impact on Akt phosphorylation by causing internalization and degradation of total IGF-1R, and by competing with IGF-1 and IGF-2 for binding to IGF-1R [25]. This in turn results in decreased IGF-1 signaling, decreased p-IGF-1R, decreased p-IRS-1, and decreased p-Akt. Although many receptor tyrosine kinases (RTK) signal through the Akt pathway, treatment with R1507 specifically inhibits signaling through IGF-1R since R1507 does not cross-react with other receptors. The decrease in p-Akt after treatment with increasing concentrations of R1507 reflects the change in phosphorylation due to increasing IGF-1R inhibition, and is dose-dependent (Figure 4c). Residual Akt phosphorylation seen after R1507 treatment may be due to other RTKs, whereas phosphorylation of IGF-1R is inhibited completely at the lowest dose of R1507 due to its specificity. MAP kinase signaling was not inhibited by R1507 or EWS/FLI-1 siRNA, however, p-ERK 1/2 levels increased after blocking the IGF-1 pathway with R1507 or EWS/FLI-1 siRNA, perhaps as a compensatory signaling pathway. Hence both agents work to inhibit the proliferation of Ewing's sarcoma cells by inhibiting phosphorylation of Akt, albeit via different upstream mechanisms (Figure 5).

Ewing's sarcoma cell lines bearing type 1 EWS/FLI-1 fusion proteins have been postulated to be less virulent than their type 2- and 3- expressing counterparts [32] and patients bearing the type 1 fusion protein were thought to have an improved outcome compared to those expressing other fusion protein types [33]. Interestingly, TC-32 and TC-71 cells express the type 1 fusion protein and are the most sensitive to R1507 by clonogenic assay. Similar results were found in a phase I clinical trial with AMG 479 (Amgen), another anti-IGF-1R monoclonal antibody (J Rodon, personal communication) [8]. However, the majority of Ewing's sarcoma patients express the type 1 fusion protein [33], [34], while the response rate to anti-IGF-1R antibodies is considerably lower. More recent publications have failed to confirm this association [35], [36]. Some authors suggest that variability of transcriptional targets of the different fusion proteins could account for some of the clinical differences [32]. Why some patients respond to anti-IGF-1R antibodies and others do not is much more complicated than just the type of fusion protein they harbor (Table 1).

The degree of inhibition of Ewing's sarcoma cell growth by R1507 may vary with assay conditions (K-P Kuenkele, personal communication). This may confound the preclinical assessment of how the expression pattern of various members of the IGF system in Ewing's sarcoma cell lines relate to responsiveness to R1507 and/or other IGF-1R inhibitors [10]. Indeed, total IGF-1R expression level was not predictive of a response to R1507 in our cell lines, as previously reported for a different anti-IGF-1R antibody (h7C10, Merck) in rhabdomyosarcoma and R1507 and AMG479 in osteosarcoma [37]–[39].

IGF-2R acts as a classic tumor suppressor gene in several different types of cancer [40]–[47], but its relevance in Ewing's sarcoma is unknown. Several mutations inactivate and/or down-regulate IGF-2R [16], [40], [42], [45], resulting in increased levels of IGF-2, and increased signaling through IGF-1R [40]–[47]. We did not detect these specific mutations in our cell lines. It remains unclear whether the GG SNP in the IGF-2 binding domain of IGF-2R is causally related to the higher levels of IGF-2 in TC-32 and TC-71, or if other factors are involved. This topic merits further investigation because preclinical studies have shown that several cancers expressing high levels of IGF-2 due to IGF-2R mutation and loss of heterozygosity (LOH) are more responsive to both anti-IGF-1R antibodies and anti-sense directed against IGF-2 or IGF-1R [43]–[46]. IGF-2R gene status has also been used to determine the course of treatment in head and neck cancer [42].

In conclusion, both EWS/FLI-1 siRNA and R1507 inhibit cellular proliferation by targeting a common pathway, namely the down-regulation of p-IGF-1R and p-Akt (Figure 5). The involvement of this pathway may explain why some patients with Ewing's sarcoma demonstrate marked responses to anti-IGF-1R antibodies [4]–[9]. However, a limitation of the hypothesis presented in Figure 5 is that different cell lines and/or patients may demonstrate divergent signaling. Our data is consistent with that of previously published studies demonstrating down-regulation of p-Akt in sarcoma xenografts [38], [39]. EWS/FLI-1 siRNA appears to have more apoptotic activity than R1507 because it (siRNA) up-regulates IGF-BP3, which can also induce apoptosis independently of the IGF system, perhaps through nucleo-mitochondrial translocation of RXRα/Nur77 [48]. Further studies are needed to elucidate resistance mechanisms since not all Ewing's sarcoma patients are responsive. These resistance mechanisms may involve differences in various parts of the IGF/IGFR signaling complex, and may also include the inactivation of tumor suppressor genes including p53, WT1, and PTEN. Alternatively, resistance to anti-IGF-1R treatment in Ewing's sarcoma could be related to the activation of alternate signaling pathways including EGFR and IR [10], [26]–[28]. Understanding mechanisms of activity, resistance, and the role of combination therapy, including conventional agents or newer molecules that target signals downstream of IGF/IGFR or that interfere with EWS/FLI-1 function [49], [50], will be paramount to improving on the promise of success seen thus far.

## Supporting Information

Table S1 Primers used for amplification of exons 27–40 of IGF-2R.
(TIF)
