# Supplementary figures and images for: R1507, an Anti-Insulin-Like Growth Factor-1 Receptor (IGF-1R) Antibody, and EWS/FLI-1 siRNA in Ewing's Sarcoma: Convergence at the IGF/IGFR/Akt Axis
Source: PLoS One. 2011 Oct 11;6(10):e26060. doi: 10.1371/journal.pone.0026060 (PMC3191161; doi:10.1371/journal.pone.0026060)

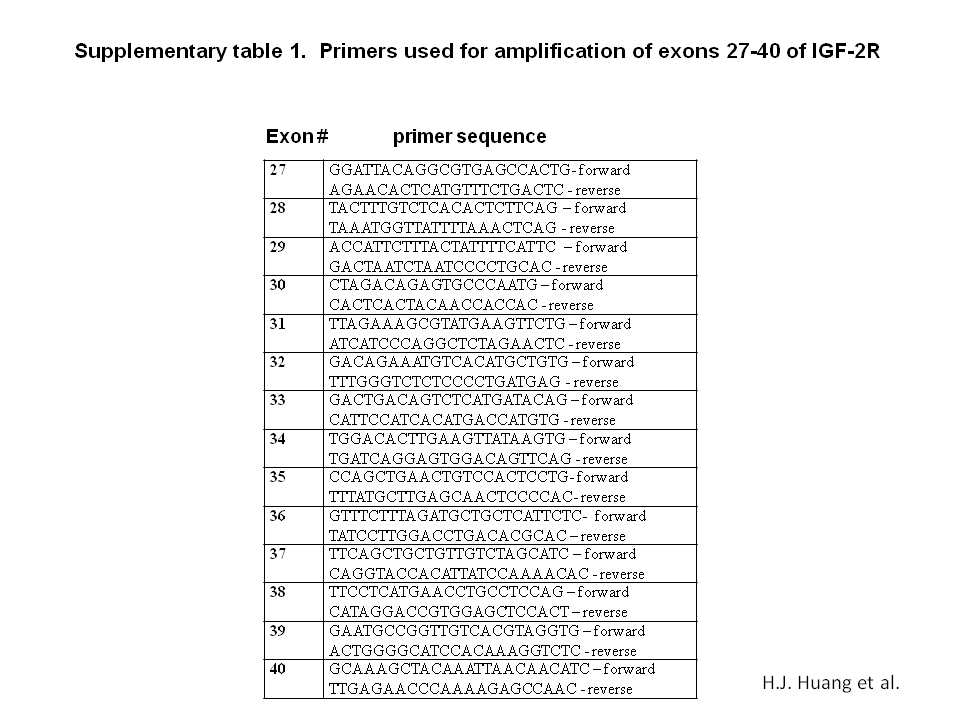

Supplement: Table S1 — Primers used for amplification of exons 27–40 of IGF-2R. (TIF) [file pone.0026060.s001.tif]
